# Clinical Decision Support to Increase Emergency Department Naloxone Coprescribing: Implementation Report

**DOI:** 10.2196/58276

**Published:** 2024-11-06

**Authors:** Stuart W Sommers, Heather J Tolle, Katy E Trinkley, Christine G Johnston, Caitlin L Dietsche, Stephanie V Eldred, Abraham T Wick, Jason A Hoppe

**Affiliations:** 1 Department of Emergency Medicine, School of Medicine, University of Colorado Anschutz Medical Campus, 12401 East 17th Avenue, 7th Floor, Aurora, CO, 80045, United States, 1 2039821107; 2 Adult and Child Center for Outcomes Research and Delivery Science Center, University of Colorado Anschutz Medical Campus, Aurora, CO, United States; 3 Department of Family Medicine, School of Medicine, University of Colorado Anschutz Medical Campus, Aurora, CO, United States; 4 Department of Medicine-Internal Medicine, School of Medicine, University of Colorado Anschutz Medical Campus, Aurora, CO, United States; 5 Department of Medicine-Hospital Medicine, School of Medicine, University of Colorado Anschutz Medical Campus, Aurora, CO, United States; 6 Information Technology Epic Application Systems, UCHealth Metro Denver, Aurora, CO, United States; 7 Department of Emergency Medicine-Medical Toxicology and Pharmacology, School of Medicine, University of Colorado Anschutz Medical Campus, Aurora, CO, United States

**Keywords:** clinical decision support systems, order sets, drug monitoring, opioid analgesic, opioid use, opioid prescribing, drug overdose, opioid overdose, naloxone, naloxone coprescribing, harm reduction, harm minimization

## Abstract

**Background:**

Coprescribing naloxone with opioid analgesics is a Centers for Disease Control and Prevention (CDC) best practice to mitigate the risk of fatal opioid overdose, yet coprescription by emergency medicine clinicians is rare, occurring less than 5% of the time it is indicated. Clinical decision support (CDS) has been associated with increased naloxone prescribing; however, key CDS design characteristics and pragmatic outcome measures necessary to understand replicability and effectiveness have not been reported.

**Objective:**

This study aimed to rigorously evaluate and quantify the impact of CDS designed to improve emergency department (ED) naloxone coprescribing. We hypothesized CDS would increase naloxone coprescribing and the number of naloxone prescriptions filled by patients discharged from EDs in a large health care system.

**Methods:**

Following user-centered design principles, we designed and implemented a fully automated, interruptive, electronic health record–based CDS to nudge clinicians to coprescribe naloxone with high-risk opioid prescriptions. “High-risk” opioid prescriptions were defined as any opioid analgesic prescription ≥90 total morphine milligram equivalents per day or for patients with a prior diagnosis of opioid use disorder or opioid overdose. The Reach, Effectiveness, Adoption, Implementation, and Maintenance (RE-AIM) framework was used to evaluate pragmatic CDS outcomes of reach, effectiveness, adoption, implementation, and maintenance. Effectiveness was the primary outcome of interest and was assessed by (1) constructing a Bayesian structural time-series model of the number of ED visits with naloxone coprescriptions before and after CDS implementation and (2) calculating the percentage of naloxone prescriptions associated with CDS that were filled at an outpatient pharmacy. Mann-Kendall tests were used to evaluate longitudinal trends in CDS adoption. All outcomes were analyzed in R (version 4.2.2; R Core Team).

**Implementation (Results):**

Between November 2019 and July 2023, there were 1,994,994 ED visits. CDS reached clinicians in 0.83% (16,566/1,994,994) of all visits and 15.99% (16,566/103,606) of ED visits where an opioid was prescribed at discharge. Clinicians adopted CDS, coprescribing naloxone in 34.36% (6613/19,246) of alerts. CDS was effective, increasing naloxone coprescribing from baseline by 18.1 (95% CI 17.9‐18.3) coprescriptions per week or 2,327% (95% CI 3390‐3490). Patients filled 43.80% (1989/4541) of naloxone coprescriptions. The CDS was implemented simultaneously at every ED and no adaptations were made to CDS postimplementation. CDS was maintained beyond the study period and maintained its effect, with adoption increasing over time (τ=0.454; *P*<.001).

**Conclusions:**

Our findings advance the evidence that electronic health record–based CDS increases the number of naloxone coprescriptions and improves the distribution of naloxone. Our time series analysis controls for secular trends and strongly suggests that minimally interruptive CDS significantly improves process outcomes.

## Introduction

Overdose (OD) deaths decreased in the United States from 2022 to 2023, but 81,083 people still died from opioids in 2023 [1]. Almost 10 million adults misused prescription opioids in 2019 [2], making opioids the most misused prescription drug [3]. Up to 20% of emergency department (ED) visits result in an opioid prescription, and ED opioid prescribing has been associated with increased opioid misuse, abuse, and death [4-10], underscoring the need for ED harm reduction.

Naloxone is an opioid antagonist capable of reversing opioid OD. Naloxone distribution has been associated with reductions in population-level opioid mortality [11,12]. Prescribing naloxone with opioids (naloxone coprescribing) is a Centers for Disease Control and Prevention (CDC) best practice and has been mandated in some states [13,14]. Yet, naloxone coprescribing remains rare [15-17], only occurring 2.3% of the time when >90 morphine milligram equivalents of opioids are ordered from the ED and 7.4% of the time after an ED visit for suspected opioid OD (vs epinephrine which is prescribed in 49% of ED visits for anaphylaxis) [16,17]. Stigma, workload, and time pressures may explain these gaps [18-21].

Health systems have begun implementing strategies to facilitate naloxone coprescribing [22]. Computerized clinical decision support (CDS) is a strategy to assist decision-making and improve health care quality [23,24]. When designed well, CDS have been shown to improve evidence-based prescribing [25-27], as well as opioid OD education and naloxone distribution [28-33]. CDS best practices include increasing specificity and sensitivity, triggering at the right time, making the evidence-based choice the easiest option, and tracking patient outcomes [24,34,35]. Effective CDS implementation requires attention to choice architecture, setting, and best practices to reduce bias and improve adoption [36-38].

We aimed to improve the evidence-based delivery of naloxone by developing and deploying an ED clinician-facing, electronic health record (EHR)–based CDS. We quantified the impact of CDS according to the Reach, Effectiveness, Adoption, Implementation, and Maintenance (RE-AIM) framework [39]. By specifying the users targeted, including workflow events that triggered CDS, and describing lessons learned, we hope to encourage the deployment and testing of similar CDS beyond our health system.

## Methods

### Intervention

Following user-centered design (UCD) principles [40], a multidisciplinary team including 5 physicians, 2 pharmacists, and several EHR builders, with expertise in implementation science, informatics, behavioral economics, and health services research, designed a fully automated, EHR-embedded, interruptive, provider-facing CDS. The intervention was beta-tested by several ED clinicians in a practice setting for 6 months before the systemwide rollout. CDS did not interface with any technologies beyond the EHR and fired within typical workflow to recommend and facilitate the addition of a naloxone prescription before the e-signing of any high-risk opioid analgesic prescription order (Multimedia Appendix 1). High-risk criteria were adapted from the 2016 CDC guidelines for chronic pain and defined as any opioid prescription (1) resulting in >90 morphine milligram equivalents per day, (2) for a patient with an opioid use disorder (OUD) diagnosis, or (3) prior opioid OD [41]. CDS searched for Systematized Nomenclature of Medicine Clinical Terms in the “Problem List Diagnosis,” “Encounter Diagnosis,” and “Hospital Problem Diagnosis” lists. Alerts were suppressed if the patient had an active naloxone prescription or if the patient was discharged to hospice, given patients on end-of-life care are excluded from CDC guidelines [41]. Naloxone prescriptions stayed on the patient’s medication list for 1 year.

Key design principles followed were that CDS be intuitive, trigger only when indicated, and default to a preselected naloxone order that was the least expensive option in the health care system’s retail pharmacies [24,34,35]. Any provider with prescribing privileges could encounter the alert. Default selection was chosen to decrease work (clicking “Accept” added naloxone to the existing order) and because “opt-out” approaches increase the uptake of target clinical behaviors [42-45].

Accepting CDS was the path of least resistance. However, consistent with nudge theory, clinicians could bypass CDS by (1) selecting “Do Not Order” then “Accept” (2 clicks); (2) selecting prepopulated bypass options (“Doesn’t meet criteria,” going to “Hospice/SNF,” “Already has naloxone”) then “Accept” (2 clicks);” or (3) commenting (≥2 clicks) [46]. “Already has naloxone” was included to account for naloxone outside the EHR. Clinicians were returned to their prior workflow after any action.

Clinicians were educated on CDS via departmental meetings and email. Educational materials included (1) CDS rationale, (2) instructions for use, and (3) suggested patient communication. No ongoing education was provided, and no changes were made to CDS after implementation.

### Study Design and Setting

This was a retrospective, observational study of ED visits in a large, not-for-profit university-affiliated, nongovernmental health care system. Located in the Rocky Mountain Region, the system has >500,000 total ED visits per year and includes 12 EDs—1 urban-academic level 1 trauma center, 2 urban community hospitals (1 a level 1 trauma center), 2 suburban community level 2 trauma centers, and 7 community free-standing EDs. The study was approved and informed consent was waived by the Colorado Multiple Institutional Review Board (COMIRB). The Guidelines and Checklist for the Reporting on Digital Health Implementations (iCHECK-DH) were followed (Multimedia Appendix 2) [47].

### Data Collection, Measurements, and Outcomes

Naloxone coprescribing was defined as a clinician prescribing opioids and naloxone during the same ED visit. Deidentified patient characteristics (age, sex, race, ethnicity, preferred language, and insurance), CDS data (reasons for firing, number of firings per visit, clinician actions, and bypass reasons), and clinical variables (whether naloxone was prescribed via CDS and the prescription was filled) were extracted monthly from the shared EHR (Epic Systems). Research data governance was linked to EHR data governance. Clinicians entered data into Epic Hyperspace and CDS responses were automatically registered in real time. Extract, transform, and load processes transferred all patient data into relational databases hosted on private virtually protected servers nightly, and a Microsoft SQL Server Management Studio query was run to further clean and filter research data into Microsoft Excel.

The RE-AIM framework was used to determine the impact of CDS [39]. More explicitly, reach was measured by examining the proportion of ED visits where CDS was triggered and whether patients’ characteristics influenced opioid prescribing (and high-risk opioid prescribing, ie, CDS triggering) and naloxone coprescribing. Effectiveness (primary outcome) was assessed by evaluating the number of ED discharges with naloxone coprescriptions per week across the system before and after CDS implementation. Effectiveness was also measured by quantifying the naloxone prescription fill rate (naloxone prescription fills per naloxone orders via CDS vs other workflows) at a 24-hour ED outpatient retail pharmacy in the largest urban academic ED. This subgroup analysis was performed to determine whether increased naloxone orders translated to more naloxone reaching patients and to compare whether patients prescribed naloxone via CDS were more likely to fill their prescriptions than patients prescribed naloxone via other workflows. All prescriptions written at this ED defaulted to the ED’s outpatient pharmacy—unless specifically requested by the patient—thus prescription fill data were available in the pharmacy EHR. Adoption was defined as the number of naloxone prescriptions from CDS per number of CDS firings. Due to EHR limitations, we could not measure CDS suppression. The process of implementation is described. Finally, maintenance was judged by whether CDS was maintained after the study period and by modeling changes in adoption over time.

### Ethical Considerations

All data releases were cleared by a Research Services Manager who ensured the data being released were compliant with the Health Insurance Portability and Accountability Act (HIPAA) and the corresponding institutional review board exemption (#23-0458). No continuing review was required because this was secondary research and all data were deidentified. Results were shared via secure email. Individual informed patient consent was waived and no compensation was offered, given no patient participation or protected health information was shared.

### Data Analysis

There is a documented need for rigorous, pragmatic evaluation when implementing new CDS [34]. Interrupted time series analyses are suggested for CDS evaluation because they control for confounding secular trends [34,48-50]. We used a Bayesian structural time-series model controlling for the number of ED visits to evaluate the impact of CDS on naloxone coprescribing (CausalImpact package; version 1.3.0; Brodersen et al) [51], Mann-Kendall tests to model longitudinal changes in CDS adoption, and chi-square tests to compare the proportions of individuals who triggered CDS and were prescribed either an opioid or opioid with naloxone across demographic categories. Equity of RE-AIM outcomes was evaluated based on patient characteristics because prior research has demonstrated an increased likelihood of opioid prescribing for White patients and increased naloxone prescribing (and coprescribing) for Black and Latine patients [52-58]. Otherwise, frequencies and percentages are reported for categorical variables. All statistical analyses were conducted in R (version 4.2.2; R Core Team) [59].

### Study Sample

All ED visits with a discharge opioid prescription between March 2013 and July 2023 were included. Effectiveness was assessed by comparing weekly aggregated counts of ED visits, opioid prescriptions, and naloxone coprescriptions between the pre- (March 2013-November 2019) and postimplementation periods (November 2019-July 2023). Adoption was assessed using only post-period data. The accuracy of synthetic control models, like the Bayesian structural time-series model used, is generally improved by including more pre-period data [51]. Therefore, we extracted enough data to provide a 2:1 pre-to post-period ratio. We did not perform a prospective power calculation. Patients younger than 18 years or older than 90 years old and those who were admitted to the hospital were excluded from both periods.

## Implementation (Results)

### Demographics

After implementation, between November 2019 and July 2023, there were 1,994,994 eligible ED discharges. Of these, 5.19% (103,606/1,994,994) included an opioid analgesic prescription and 0.83% (16,566/1,994,994) of prescriptions met high-risk criteria. Most visits included female (n=1,083,973, 54.33%), White (n=1,357,153, 68.03%), non-Latine (n=1,519,584, 76.17%), English-speaking (n=1,866,744, 93.57%), and publicly insured (n=1,146,781, 57.48%) patients (Table 1). White, non-Latine, English-speaking, privately insured patients were prescribed opioids a greater proportion of the time compared to Black, Latine, non–English-speaking, and publicly insured patients (*P*<.001).

**Table 1.** Postimplementation visit demographics stratified by visit type.

	Overall: All ED^a^ visits (n=1,994,994), n (%)	Subgroup 1: ED visits with an opioid prescription (n=103,606), n (%)	Subgroup 2: ED visits with a CDS^b^ alert and a high-risk opioid prescription (n=16,566), n (%)	Subgroup 3: ED visits with a CDS alert and a naloxone coprescription (n=3077), n (%)
**Sex**				
Female	1,083,973 (54.33)	56,260 (54.30)	8670 (52.34)	1663 (54.05)
Male	905,624 (45.39)	47,094 (45.45)	7858 (47.43)	1405 (45.66)
Other	118 (0.01)	0 (0.00)	0 (0.00)	0 (0.00)
Unknown	5279 (0.26)	252 (0.24)	38 (0.23)	9 (0.29)
**Race**				
White or Caucasian	1,357,153 (68.03)	78,174 (75.45)	12,724 (76.81)	2278 (74.03)
Black or African American	210,864 (10.57)	7066 (6.82)	1198 (7.23)	273 (8.87)
Other	420,055 (21.06)	18,106 (17.48)	2606 (15.73)	406 (13.19)
Unknown	6922 (0.35)	260 (0.25)	38 (0.23)	120 (3.90)
**Ethnicity**				
Hispanic, Latine, or Spanish origin	448,959 (22.50)	20,133 (19.43)	2637 (15.92)	565 (18.36)
Non-Hispanic, Latine, or Spanish origin	1,519,584 (76.17)	82,363 (79.50)	13,818 (83.41)	2485 (80.76)
Other	19,395 (0.97)	849 (0.82)	70 (0.42)	0 (0.00)
Unknown	7056 (0.35)	261 (0.25)	41 (0.25)	27 (0.88)
**Primary language**				
English	1,866,744 (93.57)	98,153 (94.74)	15,934 (96.18)	2936 (95.42)
Spanish	86,390 (4.33)	4130 (3.99)	437 (2.64)	98 (3.18)
Other	34,614 (1/74)	1031 (1.00)	154 (0.93)	33 (1.07)
Unknown	7246 (0.36)	292 (0.28)	41 (0.25)	10 (0.32)
**Insurance**				
Public	1,146,781 (57.48)	49,374 (47.66)	10,193 (61.53)	1937 (62.95)
Military	33,395 (1.67)	2019 (1.95)	364 (2.20)	57 (1.85)
Indigent	150,202 (7.53)	9183 (8.86)	913 (5.51)	193 (6.27)
Private or other	664,616 (33.31)	43030 (41.53)	5096 (30.76)	876 (28.47)
Unknown	0 (0.00)	0 (0.00)	0 (0.00)	0 (0.00)

a ED: emergency department.

b CDS: clinical decision support.

### Reach

CDS fired in 0.83% (16,566/1,994,994) of all ED visits. A total of 15.99% (16,566/103,606) of visits with a discharge opioid prescription met high-risk criteria and triggered CDS. CDS fired multiple times in 13.17% (2182/16,566) of visits (mean 1; median 1); ED clinicians interacted with CDS 19,246 times overall. Visits triggering CDS most often involved patients who were female (n=8670, 52.34%), White (n=12,724, 76.81%), non-Latine (n=13,818, 83.41%), spoke English (n=15,934, 96.18%), and had Medicaid or Medicare (n=10,193, 61.53%; Table 1). However, adjusting for the number of visits with an opioid prescription (a prerequisite for CDS triggering), CDS was more likely to trigger in visits with male, Latine, English speaking, and publicly insured patients (*P*<.001).

### Effectiveness

Before CDS implementation, clinicians coprescribed naloxone in 0.05% (156/318,216) of ED visits when an opioid analgesic was prescribed. After CDS implementation, ED clinicians coprescribed naloxone in 3.49% (3616/103,606) of ED visits when an opioid analgesic was prescribed. In the postimplementation period, 85.09% (3077/3616) of naloxone coprescriptions originated from CDS.

**Figure 1.**
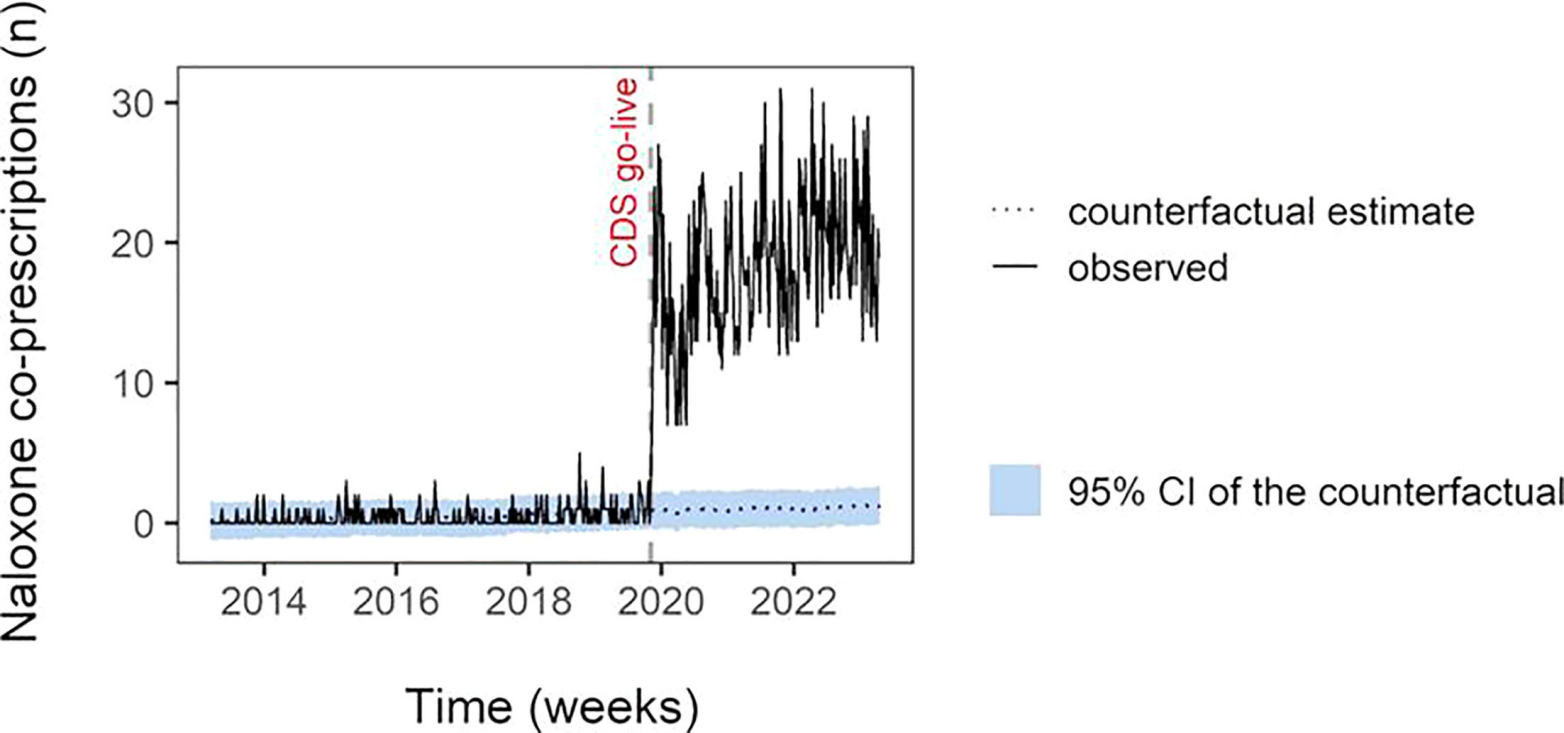
The CausalImpact plot of naloxone coprescribing. CDS: clinical decision support.

Using the number of ED visits as a covariate, the CausalImpact package predicted 0.80 (95% CI 0.55‐1.05) ED visits with naloxone coprescriptions per week and 150 (95% CI 100‐200) ED visits with naloxone coprescriptions in the postimplementation period. After CDS go-live there was an immediate increase in the number of ED visits with naloxone coprescriptions each week—18.9 ED visits with naloxone coprescriptions observed on average weekly and 3616 ED visits with naloxone coprescriptions in the entire postimplementation period (Figure 1). In other words, CDS increased ED naloxone coprescribing by 18.1 (95% CI 17.85‐18.34) naloxone coprescriptions per week or 2327% (95% CI 1702‐3335). Black and non-Latine patients were more likely to have naloxone coprescribed when CDS triggered compared to White and Latine patients (*P*<.001).

During the postimplementation period, there were 4541 naloxone coprescriptions with opioid analgesics written at the urban, academic ED (mean 1.2; median 1 per visit) and 3308 (72.85%) were ordered from a CDS alert. Patients filled 49.42% (2134/4318) of their opioid prescriptions and 43.80% (1989/4541) of naloxone coprescriptions. Patients coprescribed naloxone via CDS filled their prescriptions less often than patients coprescribed naloxone via other workflows (35.64%, 1179/3308 vs 65.69%, 810/1233).

### Adoption

ED clinicians adopted CDS, following the recommendation to coprescribe naloxone in 34.36% (6613/19,246) of alerts. Clinicians at the academic ED adopted CDS at a higher rate 61.62% (2005/3254) than at community EDs 34.70% (4608/13,280).

### Implementation

This CDS was implemented simultaneously at every ED and no changes were made to CDS postimplementation. All EDs used the same EHR, and it took a CDS builder 70 hours (including meetings, communications, and build time) to design and implement CDS.

### Maintenance

According to the Mann-Kendall test, CDS adoption increased over time (τ=0.454; *P*<.001). Because no changes were made to CDS, there were no obvious sustainability costs beyond what our health system regularly paid for EHR access. CDS is still active and currently being scaled to outpatient clinics. Sustainability decisions are made by local governance based on naloxone prescribing because it was defined as a CDC best practice. CDS are reviewed ad hoc based on technical issues and yearly otherwise.

### Lessons Learned

The implementation process benefitted from the makeup of the study team, who were able to provide local context for design, identify key workflow needs, address local barriers, and serve as champions during implementation. Beta testing and CDS-specific data analytics were prioritized to identify technical and efficiency issues early. Having data analytics built and collecting data during testing was key for providing estimates on workflow interruptiveness and informing iterative improvements. For example, monitoring revealed CDS initially only searched the current visit diagnosis, failing to identify histories of OUD and OD. The trigger algorithm was changed to include any EHR documented history of OUD or OD before going live, with a significant increase in case identification. Additionally, because clinicians told champions that CDS were firing “too late,” CDS were modified to trigger when clinicians entered as opposed to signed orders, facilitating clinician-patient communication before prescribing.

This project began as quality improvement, which was important for local buy-in. Also, the health system is funded, and therefore, owns the intervention. It would have been ideal to prospectively track implementation to elucidate system and per-patient costing and inform decisions about CDS maintenance. Future studies should formally evaluate patient-centered outcomes to confirm CDS as an effective and equitable implementation strategy.

## Discussion

### Principal Findings

A minimally interruptive CDS was readily adopted, showed a sustained effect, and significantly increased the number of ED naloxone coprescriptions. These findings support CDS as an effective implementation strategy to increase clinician uptake of naloxone best practices.

The high rate of adoption supports the need for user-centered CDS development, monitoring, and evaluation as the impact of CDS is often limited by low adoption and frequent workflow interruptions resulting in “alert fatigue” (the desensitization to important safety warnings) [24,60-64]. A Cochrane review of 122 CDS trials showed that CDS, on average, only increases the proportion of patients receiving desired care by 5.8% (95% CI 4.0% to 7.6%) [36]. The impact is variable, with the top quartile of reported improvements ranging from 10% to 62% [36]. With an adoption rate of 34.36% and a 2327% increase in the number of ED visits with naloxone coprescriptions, this CDS falls well within the top quartile of CDS improvements [36]. Interestingly, adoption increased over time. This finding differs from most other CDS literature reporting a decrease in adoption over time [65], and mirrors one other CDS study that reported a similar effect after UCD [66], perhaps suggesting that UCD improves initial and sustained adoption [66].

ED clinicians face increasingly complex workflow challenges that require validated solutions [67,68]. Previous evaluations of naloxone coprescribing CDS have not always aligned with best practices for designing, conducting, and reporting CDS interventions [28-31,34]. Prior studies have not discussed the rationale for CDS design (such as choice architecture) and have excluded key operational details (supplements and alert screenshots), making it challenging to reproduce or scale CDS [28-31,34]. The default order design of our CDS may have contributed to CDS acceptability by making choice architecture less burdensome to clinicians [34]. CDS adoption may also reflect actions in line with clinicians’ and patients’ positive attitudes toward naloxone prescribing and use [15,34,62].

Our Bayesian structural time series model, without a statutory mandate, offers robust evidence to support claims that CDS increases ED naloxone coprescribing. Our methods address the gap from prior studies that relied on pre-post designs and inferential statistics (logistic regression, *t* tests, and *χ*^2^ tests) [30,31], which increase the risk of confounding by organizational policies, regulations, or reimbursement rules [34].

The fact that White, non-Latine, English-speaking, and privately insured patients were significantly more likely to have an opioid prescribed is concerning but consistent with prior literature [52-58]. Demographic differences in opioid and naloxone prescribing have been widely reported [52-58]. It is notable that Black and non-Hispanic patients were more likely to have naloxone coprescribed after CDS was triggered. This is the first study to report demographic differences in clinicians’ responses to CDS designed to increase naloxone coprescribing. Although, Black and Latine patients are coprescribed naloxone more often at baseline. Thus, it is possible CDS increased naloxone coprescribing equally and simply failed to reduce the influence of racial and ethnic bias on opioid or naloxone prescribing [57,58]. Other CDS designers should consider these differences when implementing and evaluating CDS to ensure they do not inadvertently maintain or widen existing disparities.

### Limitations

No clinical outcomes were measured, so we do not know if practice changes impacted care such as ED readmissions. No statutory mandates were implemented during this study, but we cannot be sure local educational efforts were not made to encourage naloxone coprescribing. Larger trends in opioid prescribing were not examined but are unlikely to have impacted the rate of naloxone coprescribing.

The availability of a 24-hour ED pharmacy at the academic site was another potential operational confounder in measuring naloxone fill rates. Discharged patients had to pass the pharmacy to exit the ED. This is an important consideration for sustainability since we do not compare naloxone coprescribing versus take-home naloxone (THN). THN has been reported to improve naloxone distribution by removing the need to stop at a pharmacy and may alleviate patient costs but shift medication costs to systems or public health organizations. Prior work, evaluating THN programs, has reported naloxone distribution rates as high as 87.3% [69]. However, THN programs are resource intensive [69,70], thus, might still be improved by CDS that improve the recognition of patients at risk for OD [71].

### Conclusions

An EHR-based CDS encouraging ED naloxone coprescribing with opioid analgesics increased alert-based naloxone orders and overall system rates of naloxone coprescribing. The CDS had a low rate of interruption, a high rate of adoption [36], and significantly increased ED naloxone coprescribing across 12 EDs. There were no obvious sustainability costs beyond what the health system regularly paid for EHR access. These findings support claims that health care system leaders should consider CDS as an implementation strategy to address the significant gap in naloxone coprescribing [28-33].

## Supplementary material

10.2196/58276 Multimedia Appendix 1 Wireframe of CDS alert recommending naloxone coprescription. CDS: clinical decision support.

10.2196/58276 Multimedia Appendix 2 Completed Guidelines and Checklist for the Reporting on Digital Health Implementations (iCHECK-DH)

## Abbreviations

CDC: Centers for Disease Control and Prevention
CDS: clinical decision support
COMIRB: Colorado Multiple Institutional Review Board
ED: emergency department
HIPAA: Health Insurance Portability and Accountability Act
iCHECK-DH: Guidelines and Checklist for the Reporting on Digital Health Implementations
OD: overdose
OUD: opioid use disorder
RE-AIM: Reach, Effectiveness, Adoption, Implementation, and Maintenance
THN: take-home naloxone
UCD: user-centered design
