# Supplementary figures and images for: Clinical Decision Support to Increase Emergency Department Naloxone Coprescribing: Implementation Report
Source: JMIR Med Inform. 2024 Nov 6;12:e58276. doi: 10.2196/58276 (PMC11560079; doi:10.2196/58276)

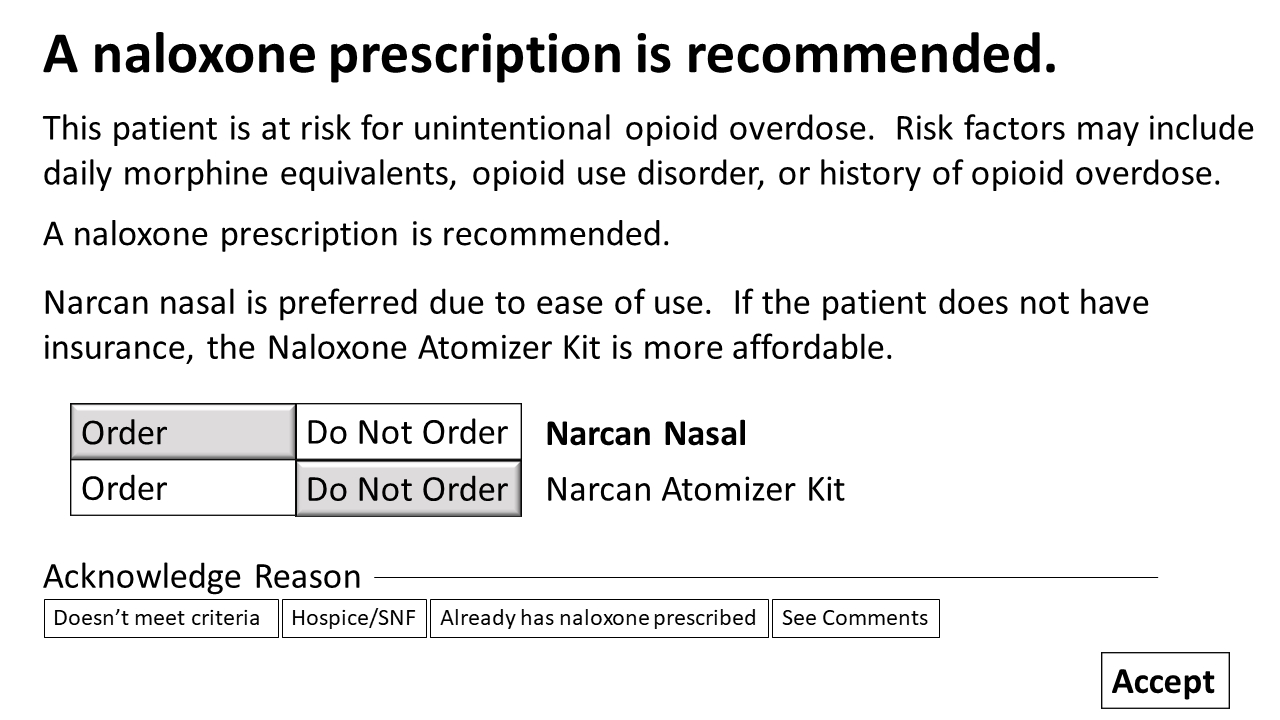

Supplement: Multimedia Appendix 1 [file medinform-v12-e58276-s001.jpg]
