# Supplementary material for: Clinical Decision Support to Increase Emergency Department Naloxone Coprescribing: Implementation Report
Source: JMIR Med Inform. 2024 Nov 6;12:e58276. doi: 10.2196/58276 (PMC11560079; doi:10.2196/58276)
Supplement: Multimedia Appendix 2 [file medinform-v12-e58276-s002.pdf]

SECTION		ITEM	DESCRIPTION	RELEVANT TEXT
**TITLE**	1	Title (M[1])	i. Identification as an implementation report, and description of the implementation in the title and/or keywords	i. Clinical Decision Support to Increase Emergency Department Naloxone Co-prescribing: Implementation Report
**ABSTRACT**	2	Abstract (M)	i. Provide a summary of the key elements of the implementation report, including a description of the implementation strategy, the intervention, defining the key elements of the implementation and health outcomes and specify the key KPIs/Outputs. We recommend describing the main aspects of the research in the following order: Background - Objectives - Methods - Implementation (Results) - Conclusions - (Optional: Trial Registration).	i. Background: Co-prescribing naloxone with opioid analgesics is a Centers for Disease Control and Prevention best practice to mitigate the risk of fatal opioid overdose (OD), yet co-prescription by emergency medicine clinicians is rare, occurring less than 5% of the time it is indicated. Clinical decision support (CDS) has been associated with increased naloxone prescribing; however, key CDS design characteristics and pragmatic outcome measures necessary to understand replicability and effectiveness have not been reported. - Objectives: This study aimed to rigorously evaluate and quantify the impact of CDS designed to improve emergency department (ED) naloxone co-prescribing. We hypothesized CDS would increase naloxone co-prescribing and the number of naloxone prescriptions filled by patients discharged from EDs in a large healthcare system. - Methods: Following user-centered design (UCD) principles, we designed and implemented a fully automated, interruptive, electronic health record (EHR)-based CDS to nudge clinicians to co-prescribe naloxone with high-risk opioid prescriptions. "High-risk" opioid prescriptions were defined as any opioid analgesic prescription ≥ 90 total morphine milligram equivalents (MMEs) per day or for patients with a prior diagnosis of opioid use disorder (OUD) or opioid overdose (OD). The RE-AIM framework was used to evaluate pragmatic CDS outcomes of *reach*, *effectiveness*, *adoption*, *implementation*, and *maintenance*. Effectiveness was the primary outcome of interest and was assessed by: 1) constructing a Bayesian structural time-series model of the number of ED visits with naloxone co-prescriptions before and after CDS implementation, and 2) calculating the percentage of naloxone prescriptions associated with CDS that were filled at an outpatient pharmacy. Mann-Kendall tests were used to evaluate longitudinal trends in CDS adoption. All outcomes were analyzed in R (version 4.2.2). - Implementation: Between 11/2019 and 7/2023, there were 1,994,994 ED visits. CDS *reached* clinicians in 0.83% (16,566/1,994,994) of all visits and 16% (16,566/103,606) of ED visits where an opioid was prescribed at discharge. Clinicians *adopted* CDS, co-prescribing naloxone in 34% (6,613/19,246) of alerts. CDS was *effective*, increasing naloxone co-prescribing from baseline by 18.1 (95% CI 17.9-18.3) co-prescriptions per week or 2,327% (95% CI 3,390-3,490). Patients filled 44% (1,989/4,541) of naloxone co-prescriptions. The CDS was *implemented* simultaneously at every ED and no adaptations were made to CDS post-

[1] M: Mandatory item

				implementation. CDS was *maintained* beyond the study period and *maintained* its effect, with adoption increasing over time (tau=0.454, *P*<.001). - Conclusions: Our findings advance the evidence that EHR-based CDS increase the number of naloxone co-prescriptions and improve the distribution of naloxone. Our time series analysis controls for secular trends and strongly suggests that minimally interruptive CDS significantly improves process outcomes.
**INTRODUCTION**	3	Context (M)	i. Describe the geographical areas, organizations, target populations and implementation context. Consider social, cultural, economic, political, health care and organizational barriers, infrastructures and facilitators that may influence implementation elsewhere. Explicitly highlight whether a national digital health strategy exists and whether implementation is aligned with the strategy.<br>ii. Describe the stage of the implementation (Developing or Adapting Solution / Piloting and Evidence generation / Package and Advocacy /Acceleration / Deploying / Scaling up / Hand over or Complete).[2]	i. Overdose (OD) deaths decreased in the U.S. from 2022 to 2023, but 81,083 people still died from opioids in 2023.[1] Almost ten million adults misused prescription opioids in 2019,[2] making opioids the most misused prescription drug.[3] Up to 20% of emergency department (ED) visits result in an opioid prescription, and ED opioid prescribing has been associated with increased opioid misuse, abuse, and death,[4-10] underscoring the critical need for ED harm reduction.<br><br>Naloxone is an opioid antagonist capable of reversing opioid OD. Naloxone distribution has been associated with reductions in population-level opioid mortality.[11-12] Prescribing naloxone with opioids (naloxone co-prescribing) is a Centers for Disease Control and Prevention (CDC) best practice and has been mandated in some states.[13-14].<br>ii. We aimed to improve evidence-based delivery of naloxone by developing and deploying an ED clinician-facing, electronic health record (EHR)-based CDS.
	4	Problem statement (M)	i. Description of the health care or public health problem challenge, or deficiency that the implementation aims to address (If applicable, include a reference to the 'health system challenge of the WHO Classification of Digital Health Interventions[3] in the description)	i. Naloxone is an opioid antagonist capable of reversing opioid OD. Naloxone distribution has been associated with reductions in population-level opioid mortality.[11-12] Prescribing naloxone with opioids (naloxone co-prescribing) is a Centers for Disease Control and Prevention (CDC) best practice and has been mandated in some states.[13-14] Yet, naloxone co-prescribing remains rare.[15-17] Naloxone is co-prescribed 2.3% of the time >90 morphine milligram equivalents (MME) of opioids are ordered from the ED and 7.4% of the time after an ED visit for suspected opioid OD (versus epinephrine which is prescribed in 49% of ED visits for anaphylaxis).[16-17] Stigma, workload, and time pressures may explain these gaps.[18-21]
	5	Similar Interventions (M)	i. Mention whether this implementation was inspired by another existing one, and if so, what is the added value of your intervention, if any, compared to the initial one? And what, if anything, has been done differently?	*** Satisfied by text in both the introduction and discussion sections<br>i. When designed well, CDS have been shown to improve evidence-based prescribing,[25-27] as well as opioid OD education and naloxone distribution.[28-33]<br>ii. Previous evaluations of naloxone co-prescribing CDS have not always aligned with best practices for designing, conducting, and reporting CDS interventions.[28-31, 34] Prior

[2] *Stages adapted from WHO Digital Health Atlas*

[3] https://apps.who.int/iris/bitstream/handle/10665/260480/WHO-RHR-18.06-eng.pdf?sequence=1&isAllowed=y

				studies have not discussed the rationale for CDS design (such as choice architecture) and have excluded key operational details (supplements and alert screenshots), making it challenging to reproduce or scale CDS.[28-31, 34]
**METHODS**	6	Aims and Objectives (M)	i. Describe the main objectives and the overall aim of the implementation.<br>ii. Describe how these will be measured using predefined primary and secondary outcome(s) and key performance indicators for this implementation and the expected intervention(s). *For example: indicators or proxy-indicators measuring direct health outcomes (e.g., HbA1c for diabetic patients); Key Performance Indicators (e.g., number of users, number of users that are properly trained, user satisfaction); Indicator assessing a particular process (e.g., administrative time for patient admission);* (If there was no evaluation, provide detailed explanation for reasoning)	***Satisfied by text in both the introduction and methods sections<br>i. We aimed to improve evidence-based delivery of naloxone by designing developing and implementing deploying an electronic health record (EHR)-based CDS. We quantified the impact of CDS according to the Reach, Effectiveness, Adoption, Implementation, and Maintenance (RE-AIM) framework.[39] By specifying the users targeted, including workflow events that triggered CDS, and describing lessons learned, we hope to encourage deployment and testing of similar CDS beyond our health system.<br>ii. The RE-AIM framework was used to determine the impact of CDS.[39] More explicitly, reach was measured by examining the proportion of ED visits where CDS triggered and whether patients' characteristics influenced opioid prescribing (and high-risk opioid prescribing, i.e., CDS triggering) and/or naloxone co-prescribing. Effectiveness (primary outcome) was assessed by evaluating the number of ED discharges with naloxone co-prescriptions per week across the system before and after CDS implementation. Effectiveness was also measured by quantifying the naloxone prescription fill rate (naloxone prescription fills per naloxone orders via CDS versus other workflows) at a 24-hour ED outpatient retail pharmacy in the largest urban-academic ED. This subgroup analysis was performed to determine whether increased naloxone orders translated to more naloxone reaching patients and to compare whether patients prescribed naloxone via CDS were more likely to fill their prescription than patients prescribed naloxone via other workflows. This analysis focused on one site because all prescriptions written at this ED defaulted to the ED's outpatient pharmacy—unless specifically requested by the patient—thus prescription fill data were available in the pharmacy EHR. Adoption was defined as the number of naloxone prescriptions from CDS per number of CDS firings. Due to EHR limitations, we could not measure CDS suppression. The process of implementation is described. Finally, maintenance was judged by whether CDS was maintained after the study period and by modelling changes in adoption over time.
	7	Blueprint summary (M)	i. Describe the design and key features of the intervention and key points of the implementation strategy and roadmap.	i. Following user-centered design (UCD) principles,[40] a multidisciplinary team including five physicians, two pharmacists, and several EHR builders, with expertise in implementation science, informatics, behavioral economics, and health services research, designed a fully automated, EHR embedded, interruptive, provider-facing CDS. The intervention was beta-tested by several ED clinicians in a practice setting for six months before systemwide rollout…. [After rollout] Clinicians were educated on CDS via departmental meetings and email. Educational materials included 1) CDS

			rationale, 2) instructions for use, and 3) suggested patient communication. No ongoing education was provided, and no changes were made to CDS after implementation…. This was a retrospective, observational study of ED visits in a large, not-for-profit university-affiliated, nongovernmental healthcare system. Located in the Rocky Mountain Region, the system has >500,000 total ED visits per year and includes 12 EDs: one urban-academic level 1 trauma center, two urban community hospitals (one a level 1 trauma center), two suburban community level 2 trauma centers, and 7 community free-standing EDs. The study was approved and informed consent was waived by the Colorado Multiple Institutional Review Board (COMIRB). The Guidelines and Checklist for the Reporting on Digital Health Implementations (iCHECK-DH) were followed.[47]
8	Technical Design (M)	i. Reasons for developing or choosing this tool. Does it combine several tools? Provide a brief description of the tool(s) (functionality and architecture) and how it fits into the health enterprise architecture and investment roadmap (if applicable). Indicate whether the solution is based on an existing solution or has been developed or purchased specifically for this intervention.<br>Describe the type of technology used (e.g., AI applications), license of the technology (open source, free, commercial, IP ownership etc.), include code documentation (if available), link to the application, link to wiki or project website.	i. CDS did not interface with any technologies beyond the EHR and fired within typical workflow to recommend and facilitate the addition of a naloxone prescription before the e-signing of any high-risk opioid analgesic prescription order (Multimedia Appendix 1: Wireframe of CDS alert recommending naloxone co-prescription). High-risk criteria were adapted from the 2016 CDC guidelines for chronic pain and defined as any opioid prescription: 1) resulting in > 90 MMEs per day, 2) for a patient with an opioid use disorder (OUD) diagnosis, and/or 3) prior opioid OD. [41] CDS searched for CDS searched for Systematized Nomenclature of Medicine Clinical Terms (SNOMED CT) diagnostic codes in the "Problem List Diagnosis,", "Encounter Diagnosis,", and "Hospital Problem Diagnosis" lists. Alerts were suppressed if the patient had an active naloxone prescription or if the patient was discharged to hospice, given patients on end-of-life care are excluded from CDC guidelines [41]. Naloxone prescriptions stayed on the patient's medication list for one1 year.<br><br>Key design principles followed were that CDS be intuitive, trigger only when indicated, and default to a preselected naloxone order that was the least expensive option in our healthcare system's retail pharmacies.[24, 34-35] Any provider with prescribing privileges could encounter the alert. Default selection was chosen to decrease work (clicking "Accept" added naloxone to the existing order) and because "opt-out" approaches increase the uptake of target clinical behaviors.[42-45]<br><br>Accepting CDS was the path of least resistance. However, consistent with nudge theory, clinicians could bypass CDS by: 1) select "Do Not Order" then "Accept" (2 clicks), 2) selecting prepopulated bypass options ("Doesn't meet criteria," going to "Hospice/SNF," "Already has naloxone") then "Accept" (2 clicks)," or 3) commenting (>2 clicks).[46] "Already has naloxone" was included to account for naloxone outside the EHR. Clinicians were returned to their prior workflow after any action.

9	Target (M)	i.	The target refers to the focus or recipient of the intervention. It is the specific person, group, system, or problem that the intervention aims to change or improve. The characteristics of the targeted "site(s)" (locations, staff, resources, etc.) for implementation and any eligibility criteria. The population targeted by the intervention and any eligibility criteria.	i.	Any provider with prescribing privileges could encounter the alert…. This was a retrospective, observational study of ED visits in a large, not-for-profit university-affiliated, nongovernmental healthcare system. Located in the Rocky Mountain Region, the system has >500,000 total ED visits per year and includes 12 EDs: one urban-academic level 1 trauma center, two urban community hospitals (one a level 1 trauma center), two suburban community level 2 trauma centers, and 7 community free-standing EDs.
10	Data (M)	i.	Describe the data governance, including life cycle (collection, processing, storage, modification, sharing, suppression), the data ownership (mention whether patients actually have access to the data), data protection measures, confidential use of routine data, expected level of data integration, data for research, cross-border data agreement, if any, the applicable legal framework, and how the project complies with it. Data consent: Has patient consent been obtained? Describe the approach to data protection and cybersecurity (e.g. security by design, privacy by design, etc.) and where the data is hosted. (e.g., in-country, cloud based, hybrid model etc.). Describe, if applicable, the government preferences in terms of data policies.	i.	Deidentified patient characteristics (age, sex, race, ethnicity, preferred language, insurance), CDS data (reasons for firing, number of firings per visit, clinician actions, bypass reasons), and clinical variables (whether naloxone was prescribed via CDS and/or the prescription was filled) were extracted monthly from the shared EHR (Epic Systems; Verona, WI). Research data governance was linked to EHR data governance. Clinicians entered data into Epic Hyperspace and CDS responses were automatically registered in real-time. Extract, Transform, and Load (ETL) processes transferred all patient data into relational databases hosted on private virtually protected servers nightly, and a Microsoft SQL Server Management Studio query was run to further clean and filter research data into Microsoft Excel….All data releases were cleared by a Research Services Manager who ensured the data being released were compliant with the Health Insurance Portability and Accountability Act (HIPAA) and the corresponding Institutional Review Board (IRB) exemption (#23-0458). No continuing review was required because this was secondary research and all data were deidentified. Results were shared via secure email. Individual informed patient consent was waived and no compensation was offered, given there was no patient participation or protected health information shared.
11	Interoperability (M)	i.	Describe the interfaces (what other systems does the tool connect to) and the standards that were used (which specific ones and rationale of choice) (e.g., semantic ontologies such ICD as SNOMED, LOINC or technical standards such as HL7 FHIR, etc.).		CDS did not interface with any technologies beyond the EHR and fired within typical workflow to recommend and facilitate the addition of a naloxone prescription before the e-signing of any high-risk opioid analgesic prescription order (Multimedia Appendix 1: Wireframe of CDS alert recommending naloxone co-prescription). High-risk criteria were adapted from the 2016 CDC guidelines for chronic pain and defined as any opioid prescription: 1) resulting in > 90 MMEs per day, 2) for a patient with an opioid use disorder (OUD) diagnosis, and/or 3) prior opioid OD. [41] CDS searched for Systematized Nomenclature of Medicine Clinical Terms (SNOMED CT) diagnostic codes in the "Problem List Diagnosis,", "Encounter Diagnosis,", and "Hospital Problem Diagnosis" lists. Alerts were suppressed if the patient had an active naloxone prescription or if the patient was discharged to hospice, given patients on end-of-life care are excluded from CDC guidelines [41]. Naloxone prescriptions stayed on the patient's medication list for one year.

				Key design principles followed were that CDS be intuitive, trigger only when indicated, and default to a preselected naloxone order that was the least expensive option in our healthcare system's retail pharmacies.[24, 34-35] Any provider with prescribing privileges could encounter the alert. Default selection was chosen to decrease work (clicking "Accept" added naloxone to the existing order) and because "opt-out" approaches increase the uptake of target clinical behaviors.[42-45]<br><br>Accepting CDS was the path of least resistance. However, consistent with nudge theory, clinicians could bypass CDS by: 1) select "Do Not Order" then "Accept" (2 clicks), 2) selecting prepopulated bypass options ("Doesn't meet criteria," going to "Hospice/SNF," "Already has naloxone") then "Accept" (2 clicks)," or 3) commenting (>2 clicks).[46] "Already has naloxone" was included to account for naloxone outside the EHR. Clinicians were returned to their prior workflow after any action.
12	Participating entities (M)	i.	Describe the implementing organization(s): Type of organisation(s), mission, leadership, vision, etc.<br><br>Government involvement: Describe whether the government was involved in the implementation, at what level and at what stage(s).<br><br>Partners: Describe all partners (organisations) and their role in the implementation.<br><br>Funders: List all actors and stakeholders who have funded or invested in the development of the implementation (if different from the implementation, e.g. using an existing digital health intervention). Indicate their level of involvement in terms of funding.<br><br>Mention which entity will own the final product and intellectual property after the implementation phase.	i. This was a retrospective, observational study of ED visits in a large, not-for-profit university-affiliated, nongovernmental healthcare system. Located in the Rocky Mountain Region, the system has >500,000 total ED visits per year and includes 12 EDs: one urban-academic level 1 trauma center, two urban community hospitals (one a level 1 trauma center), two suburban community level 2 trauma centers, and 7 community free-standing EDs…. This project began as quality improvement, which was important for local buy-in. Also, the health system funded, and therefore, owns the intervention. It would have been ideal to prospectively track implementation to elucidate system and per patient costing and inform decisions about CDS maintenance. Future studies should formally evaluate patient-centered outcomes to confirm CDS as an effective and equitable implementation strategy.
13	Budget Planning (M)	i.	Describe the planned budget for implementation (include costs such as change management, user training, project management, technology pricing, total cost of ownership). If possible, include actual costs, otherwise describe the range or percentage of the total budget. Indicate the period covered by the budget. Describe the budget for the intervention (e.g. development, purchase or adaptation of a free tool); if possible	***There was no formal budget. However,<br>i. it took a CDS builder 70 hours (including meetings, communications, and build time) to design and implement CDS…. there were no obvious sustainability costs beyond what our health system regularly paid for EHR access.

			include real costs, otherwise describe as a percentage of the total budget. Indicate the duration covered by the budget.	
	14	Sustainability (M)	i. Describe the Business model including the sustainability model (financial, economic, environment etc.). If possible, put outcomes in relation to cost to assess sustainability. Describe long term exit strategies, and all dimensions considered to sustain the project after the end of funding. If applicable, describe potential institutionalization of the project.	ii. it took a CDS builder 70 hours (including meetings, communications, and build time) to design and implement CDS…. there were no obvious sustainability costs beyond what our health system regularly paid for EHR access.
**RESULTS**	15	Coverage (M)	i. Describe whether the coverage of implementation is international, national, regional or at the level of e.g. municipalities. If coverage is sub-national, describe the regions. Provide information on the relative importance of the coverage (e.g. % of eligible population covered).	***The coverage was described as regional and the region was described in the methods section.<br>i. CDS fired in 0.83% (16,566/1,994,994) of all ED visits. Sixteen percent (16,566/103,606) of visits with a discharge opioid prescription met high-risk criteria and triggered CDS. CDS fired multiple times in 13.2% (2,182/16,566) of visits (mean 1, median 1); ED clinicians interacted with CDS 19,246 times overall. Visits triggering CDS most often involved patients who were female (52%), White (77%), non-Latine (84%), spoke English (96%), and had Medicaid or Medicare (62%) (Table 1). However, adjusting for the number of visits with an opioid prescription (a prerequisite for CDS triggering), CDS was more likely to trigger in visits with male, Latine, English speaking, publicly insured patients (P values <.001).
	16	Outcomes (M)	i. Primary and other outcome(s) of the implementation. Detail the actual outcomes, using the pre-defined outcome measures (if applicable).	i. Reach<br>CDS fired in 0.83% (16,566/1,994,994) of all ED visits. Sixteen percent (16,566/103,606) of visits with a discharge opioid prescription met high-risk criteria and triggered CDS. CDS fired multiple times in 13.2% (2,182/16,566) of visits (mean 1, median 1); ED clinicians interacted with CDS 19,246 times overall. Visits triggering CDS most often involved patients who were female (52%), White (77%), non-Latine (84%), spoke English (96%), and had Medicaid or Medicare (62%) (Table 1). However, adjusting for the number of visits with an opioid prescription (a prerequisite for CDS triggering), CDS was more likely to trigger in visits with male, Latine, English speaking, publicly insured patients (P values <.001).<br><br>Effectiveness<br>Before CDS implementation, clinicians co-prescribed naloxone in 0.05% (156/318,216) of ED visits when an opioid analgesic was prescribed. After CDS implementation, ED clinicians co-prescribed naloxone in 3.5% (3,616/103,606) of ED visits when an opioid analgesic was prescribed. In the post-implementation period, 85% (3,077/3,616) of naloxone co-prescriptions originated from a CDS alert.

Using the number of ED visits as a covariate, the CausalImpact package predicted 0.80 (95% CI 0.55-1.05) ED visits with naloxone co-prescriptions per week and 150 (95% CI 100-200) ED visits with naloxone co-prescriptions in the post-implementation period. After CDS go-live there was an immediate increase in the number of ED visits with naloxone co-prescriptions each week: 18.9 ED visits with naloxone co-prescriptions observed on average weekly and 3,616 ED visits with naloxone co-prescriptions in the entire post-implementation period (Figure 2). In other words, CDS increased ED naloxone co-prescribing by 18.1 (95% CI 17.85-18.34) naloxone co-prescriptions per week or 2,327% (95% CI 1,702-3,335). Black and non-Latine patients were more likely to have naloxone co-prescribed when CDS triggered compared to White and Latine patients (*P* values <.001). During the post-implementation period, there were 4,541 naloxone co-prescriptions with opioid analgesics written at the urban, academic ED (mean 1.2, median 1 per visit), 3,308 (73%) were ordered from a CDS alert. Patients filled 49% (2,134/4,318) of their opioid prescriptions and 44% (1,989/4,541) of naloxone co-prescriptions. Patients co-prescribed naloxone via CDS filled their prescriptions less often than patients co-prescribed naloxone via other workflows [36% (1,179/3,308) vs 66% (810/1,233)].

Adoption

ED clinicians adopted CDS, following the recommendation to co-prescribe naloxone in 34% (6,613/19,246) of alerts. Clinicians at the academic ED adopted CDS at a higher rate 62% (2,005/3,254) than at community EDs 35% (4,608/13,280).

Implementation

This CDS was implemented simultaneously at every ED and no changes were made to CDS post-implementation. All EDs used the same EHR, and it took a CDS builder 70 hours (including meetings, communications, and build time) to design and implement CDS.

Maintenance

According to the Mann Kendall test, CDS adoption increased over time (tau=0.454, *P*<.001). Because no changes were made to CDS, there were no obvious sustainability costs beyond what our health system regularly paid for EHR access. CDS is still active and currently being scaled to outpatient clinics.

17	Lessons learned (M)	i. Describe any lessons learned from the implementation experience that could be used to improve future outcomes. This could include, but is not limited to, success factors, implementation challenges or budget considerations.<br><br>Success factors: Describe factors that positively influenced the implementation (e.g. involvement of key stakeholders). Also describe contextual factors that may have positively influenced the results (e.g. new legal requirements that facilitated adoption).<br><br>Challenges to implementation: Describe challenges (process-related, such as resistance to change, but also technical). Include contextual factors that may have affected the achievement of outcomes such as an unexpected change of government, or 'opposing key players' who, despite potential participation, may hinder implementation (e.g. software companies managing regional digital health may act as barriers to innovation).<br><br>Budget: Describe whether the implementation budget was adhered to, and if not, why not. Also detail the expected operational costs (e.g. licence, maintenance, human resources, updates to in-house developments) to estimate the total cost of ownership. Include real costs, otherwise describe them as a percentage of the total budget.<br><br>What recommendations can be drawn from the lessons learned?	i. The implementation process benefitted from the makeup of the study team, who were able to provide local context for design, identify key workflow needs, address local barriers, and serve as champions during implementation. Beta testing and CDS-specific data analytics were prioritized to identify technical and efficiency issues early. Having data analytics built and collecting data during testing was key for providing estimates on workflow interruptiveness and informing iterative improvements. For example, monitoring revealed CDS initially only searched the current visit diagnosis, failing to identify histories of OUD and/or OD. The trigger algorithm was changed to include any EHR documented history of OUD/OD before go-live, with a significant increase in case identification. Additionally, because clinicians told champions that CDS were firing “too late”, CDS were modified to trigger when clinicians entered as opposed to signed orders, facilitating clinician-patient communication before prescribing.<br>This project began as quality improvement, which was important for local buy-in. Also, the health system funded, and therefore, owns the intervention. It would have been ideal to prospectively track implementation to elucidate system and per patient costing and inform decisions about CDS maintenance. Future studies should formally evaluate patient-centered outcomes to confirm CDS as an effective and equitable implementation strategy.

	18	Unintended consequences (NM[4])	i. Describe unintended consequences (positive or negative), harms or negative side-effects (if any).	i.	NM/NA
**DISCUSSION**	19	Conclusion (M)	i. Summary of the conclusions and future implications.	i.	An EHR-based CDS encouraging ED naloxone co-prescribing with opioid analgesics increased both alert-based naloxone orders and overall system rates of naloxone co-prescribing. The CDS had a low rate of interruption, a high rate of adoption,[36] and significantly increased ED naloxone co-prescribing across 12 EDs. There were no obvious sustainability costs beyond what the health system regularly paid for EHR access. These findings support claims that healthcare system leaders should consider CDS as an implementation strategy to address the significant gap in naloxone co-prescribing.[28-33]
**GENERAL**	20	General (NM)	i. If applicable, include statement(s) on regulatory approvals (including, as appropriate, ethical approval, governance approval), trial or study registration (availability of protocol), and conflicts of interest. For implementation reports with a research component, ethical approval or a waiver from an appropriate ethics committee is required. For those without a research component, ethical considerations may still be relevant, but do not necessarily require approval or waiver. Authors may consult this article[5] for further guidance on ethical considerations in their specific context"	i.	This study was approved and informed consent was waived by the Colorado Multiple Institutional Review Board (COMIRB). The author(s) received no financial support for the research, authorship, and/or publication of this article. No approval for publication was deemed necessary.

[4] NM : Non-mandatory item

[5] Eccles, M. P., Weijer, C., & Mittman, B. (2011). Requirements for ethics committee review for studies submitted to Implementation Science. Implementation science, 6(1), 1-3.
